# A novel endoscopic ultrasound system assisted by artificial intelligence for the recognition of pancreatic parenchyma and the detection of solid/cystic lesions

**DOI:** 10.1055/a-2836-6609

**Published:** 2026-05-21

**Authors:** Sho Takahashi, Tomoya Takahashi, Toshio Fujisawa, Ippei Ikoma, Yasuhisa Jimbo, Ko Tomishima, Hiroyuki Isayama

**Affiliations:** 1 Department of Gastroenterology 73362 Graduate School of Medicine, Juntendo UniversityTokyoJapan

Endoscopic ultrasound (EUS) is an essential modality for detecting pancreatic solid and cystic lesions. However, EUS skill acquisition remains difficult for trainee endoscopists 1 . To facilitate the skill acquisition of trainee endoscopists, an EUS system assisted by artificial intelligence (EUS-AI), EW10-US01 (CAD EYE; FUJIFILM Corporation, Tokyo, Japan), has recently been developed and released for clinical use 2 3 . This system provides two functions: recognition of the pancreatic parenchyma, visualized as a white cross, and detection of solid and cystic lesions, visualized as a blue box. These outputs are overlaid in real time on live EUS images with optional acoustic alerts. We report a case in which this novel system contributed to the detection of pancreatic solid lesions that had not been identified via magnetic resonance imaging (MRI).

**Fig. 1:**
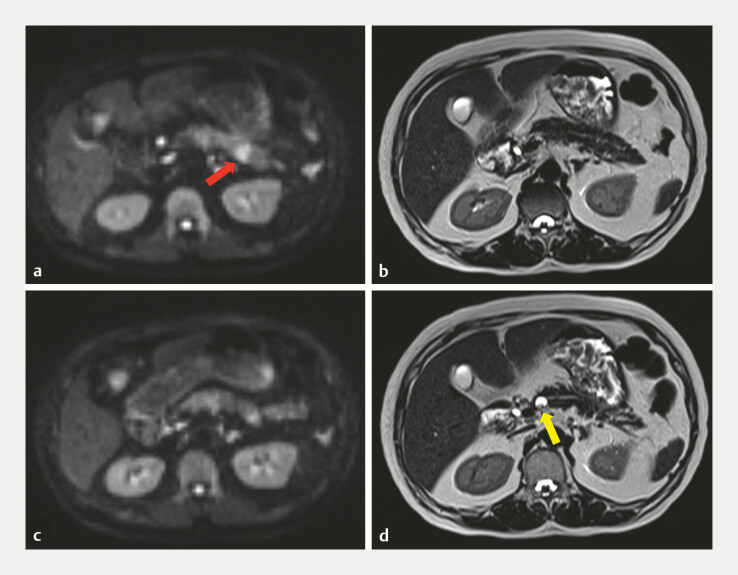
**a** Diffusion-weighted imaging (DWI) demonstrated a 15 mm hyperintense lesion in the pancreatic tail (red arrow). **b** Heavily T2-weighted imaging did not demonstrate a corresponding lesion. **c** DWI did not demonstrate a solid lesion with diffusion restriction in the pancreatic body. **d** Heavily T2-weighted imaging demonstrated a 13 mm cystic lesion in the pancreatic body (yellow arrow).

**Fig. 2:**
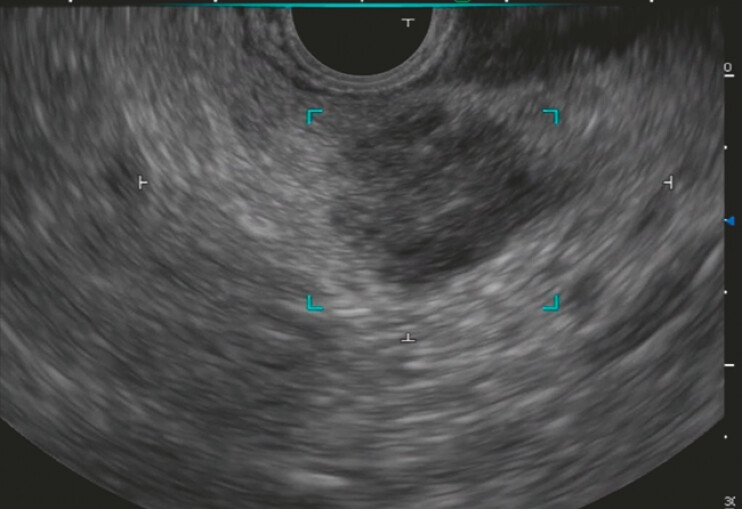
Using the EUS-AI system, the 15 mm lesion in the pancreatic tail previously detected via MRI was enclosed by the blue box. EUS-AI, EUS system assisted by artificial intelligence; MRI, magnetic resonance imaging.

**Fig. 3:**
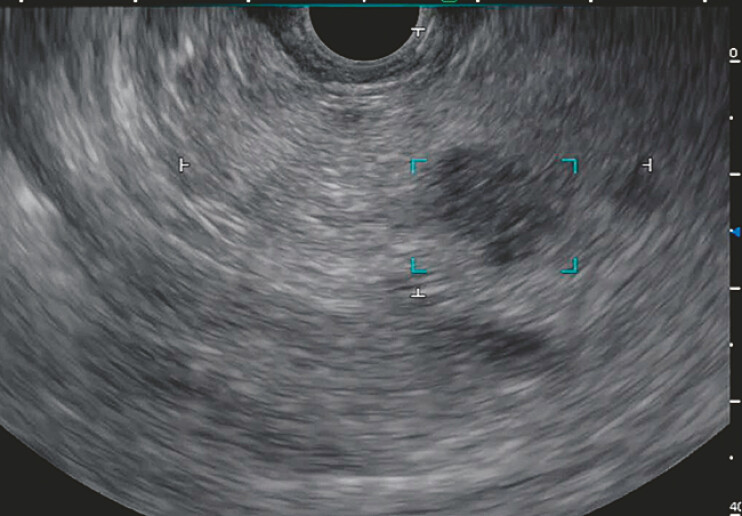
EUS-AI detected the 12 mm lesion in the pancreatic body adjacent to the cyst that had not been detected via MRI. EUS-AI, endoscopic ultrasound system assisted by artificial intelligence; MRI, magnetic resonance imaging.

An 81-year-old woman presented with worsening control of type 2 diabetes mellitus, which prompted further MRI evaluation. The examination revealed a 15 mm hyperintense lesion in the pancreatic tail in diffusion-weighted imaging ( Fig. 1 **a, b** ). In addition, a 13 mm cystic lesion in the pancreatic body was identified in heavy T2 weighted images ( Fig. 1 **c, d** ). For diagnostic confirmation, EUS-guided tissue acquisition (EUS-TA) was performed with the assistance of EUS-AI ( Video 1 ). After the previously identified lesion in the pancreatic tail had been confirmed ( Fig. 2 ), subsequent screening with EUS-AI identified another a solid 12 mm lesion adjacent to the cyst in the pancreatic body, which had not been detected via MRI ( Fig. 3 ). EUS-TA of both lesions was performed, and pathological examination confirmed adenocarcinoma. This patient underwent pancreaticoduodenectomy.

Real-time recognition of the pancreatic parenchyma and detection of solid/cystic lesions by an artificial intelligence-assisted EUS system. EUS, endoscopic ultrasound.Video 1

This case highlights the clinical feasibility of this commercially available EUS-AI system that provides the real-time recognition of pancreatic parenchyma and detection of solid and cystic lesions in routine practice. EUS-AI may contribute to more accurate examinations by facilitating the skill acquisition of trainee endoscopists.

Endoscopy_UCTN_Code_CCL_1AF_2AZ

Citation Format

Endoscopy 2026; 58: E158–E159. DOI: 10.1055/a-2776-7961
